# Experimental data on the mechanical properties of individual *caobilla* wood cubes used on control pile systems

**DOI:** 10.1016/j.dib.2018.08.045

**Published:** 2018-08-23

**Authors:** Norma Patricia López-Acosta, Eduardo Martínez-Hernández

**Affiliations:** Instituto de Ingeniería, Universidad Nacional Autónoma de México, Mexico City, Mexico

## Abstract

This article contains data on the mechanical properties of individual cubes of *caobilla* wood (*Swietenia humilis Zucc.*) used as deformable cell of control piles, which are a special foundation system used to regulate both loads and settlements in buildings. The data were obtained from stress-controlled compression tests considering wooden cubes with different volumetric weight, moisture conditions and fiber orientation. In addition, the correlation of yield stress versus volumetric weight of *caobilla* wood cubes is presented. The mean values and standard deviations of volumetric weight and yield stress of the tested wooden cubes under dry and wet states are also presented.

**Specifications Table**

**Table:** 

Subject area	*Civil Engineering, Material Science*
More specific subject area	*Physical and mechanical properties of building materials*
Type of data	*Tables and figures*
How data was acquired	*Laboratory tests*
Data format	*Raw, analyzed and tabulated*
Experimental factors	*The moisture content of the tested wooden cubes varies between*MC*= 0.5 to 1% and it represents the dry state condition in this article. Wet state condition was obtained by submerging some specimens in a container with water during 26 days before being tested*
Experimental features	*Stress-controlled compression tests in a Universal Testing Machine*
Data source location	*Materials Laboratory of the Instituto de Ingeniería, Universidad Nacional Autónoma de México, Mexico City, Mexico*
Data accessibility	*Relevant data are available within the article and some additional data are in a Supplementary data file (Appendix A)*

**Value of the data**

• The data can be used to design deformable cells of control pile systems.
• The data allow estimation of the maximum load that the piles can reach under normal working conditions, which can be compared with the capacity of the load frame of the control device to prevent its failure.
• The data allow direct estimation of the settlement of a building taking into account the deformation measured in the wooden cubes of the control devices.
• The data are useful to develop criteria for control pile maintenance.
• The data can be reanalyzed and compared with other materials used as deformable cell in order to perform additional researches.
• The dataset can be used to calculate and design new wood structural elements for buildings.
• The dataset can be reused by other researchers to investigate further properties of wooden cubes and to enable supplementary analyses.

## 1 Data

This article presents data on the mechanical properties of *caobilla* wood (*Swietenia humilis Zucc.*) cubes used in control pile systems. The data were obtained from stress-strain diagrams derived from stress-controlled compression tests (2 MPa/min) on cubes of 5 cm edge under different moisture conditions and fiber orientation (anisotropy of wood), as listed in Table 1. Table 2, Table 3, Table 4 show the yield stress *σ*_*f*_, the strain associated with the yield stress *ε*_*f*_, the ultimate stress *σ*_*m*_, the strain associated with the ultimate stress *ε*_*m*_, the tangent modulus in the elastic range *E*_*e*_ and the tangent modulus in the plastic range *E*_*p*_ of the wooden cubes. In addition, the correlation of yield stress versus volumetric weight of the tested cubes and the main descriptive statistics of wooden cubes under dry and wet states are presented.

**Table 1:** Summary of tests performed on individual wooden cubes.

**Type of specimen**	**Type of test**	**Number of specimen**
Dry cubes	Vertically oriented fibers	5
	Horizontally oriented fibers	8
Wet cubes	Horizontally oriented fibers	4

**Table 2:** Mechanical properties of individual dry cubes of *caobilla* wood with horizontally oriented fibers.

**Volumetric weight**	***σ***_**f**_	***ε***_**f**_	***σ***_**m**_	***ε***_**m**_	***E***_**e**_	***E***_**p**_
**(kN/m**^**3**^**)**	**(MPa)**	**(%)**	**(MPa)**	**(%)**	**MPa**	**MPa**
4.62	6.9	1.5	11.3	41.5	457.6	10.5
4.78	6.7	2.0	11.4	46.1	299.1	10.5
4.88	7.5	1.5	13.3	40.2	496.9	14.7
4.98	9.2	1.3	13.8	42.2	663.8	11.1
5.59	8.9	1.7	14.0	37.5	524.9	13.9
5.83	12.1	2.7	20.6	43.5	753.9	20.1
6.57	16.2	1.6	23.0	45.2	1011.3	16.9
6.65	18.6	1.4	27.3	28.0	1330.9	36.6

Note: *σ*_f_ = yield stress, *ε*_f_ = strain associated with the yield stress, *σ*_m_ = ultimate stress, *ε*_m_ = strain associated with the ultimate stress, *E*_e_ = tangent modulus in the elastic range, *E*_p_ = tangent modulus in the plastic range.

**Table 3:** Mechanical properties of individual dry cubes of *caobilla* wood with vertically oriented fibers.

**Volumetric weight**	***σ***_**f**_	***ε***_**f**_	***σ***_**m**_	***ε***_**m**_	***E***_**e**_
**(kN/m**^**3**^**)**	**(MPa)**	**(%)**	**(MPa)**	**(%)**	**(MPa)**
5.12	34.3	1.0	41.5	3.4	3432.3
5.32	34.8	1.1	45.1	5.6	3481.3
5.56	35.3	1.2	39.5	4.2	3209.4
5.94	40.5	1.8	44.3	6.5	2531.3
6.14	40.7	1.1	57.3	3.8	4069.7

**Table 4:** Mechanical properties of individual wet cubes of *caobilla* wood with horizontally oriented fibers.

**Volumetric weight**	**MC**	***σ***_**f**_	***ε***_**f**_	***σ***_**m**_	***ε***_**m**_	***E***_**e**_	***E***_**p**_
**(kN/m**^**3**^**)**	**(%)**	**(MPa)**	**(%)**	**(MPa)**	**(%)**	**(MPa)**	**(MPa)**
4.81	58.7	3.3	1.7	9.7	44.8	240.6	10.3
5.15	47.6	4.1	2.8	13.2	47.1	170.7	13.8
6.34	42.4	6.1	2.2	17.6	46.4	340.8	17.7
6.64	36.2	8.9	2.5	20.5	44.8	497.5	16.3

Note: MC = moisture content.

## 2 Experimental design, materials, and methods

### 2.1 Description of control pile device

**Fig. 1:**
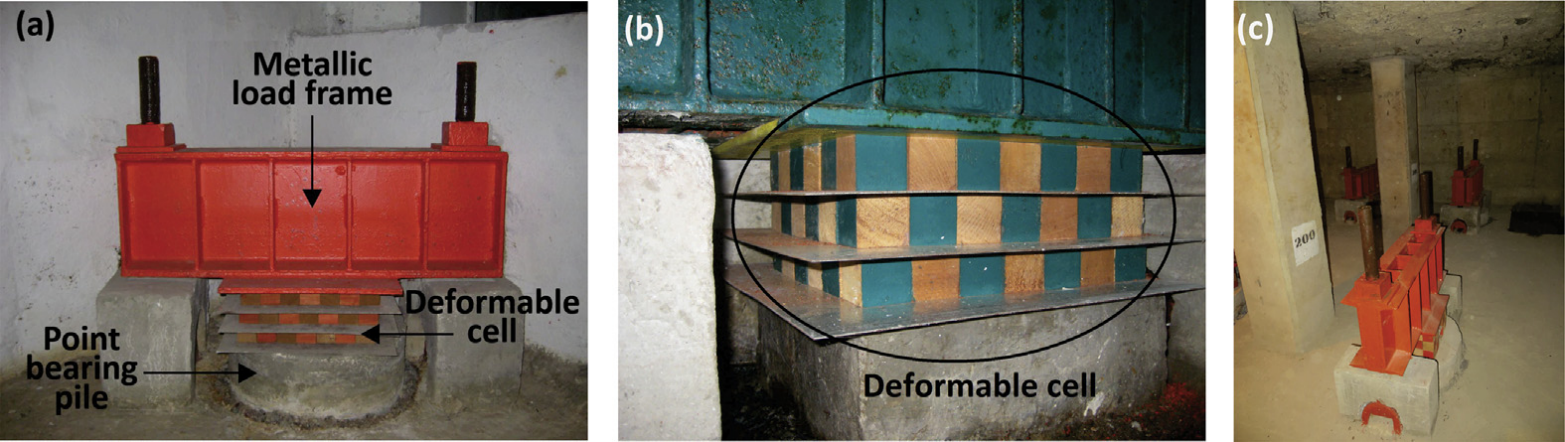
Control device: (a) individual control pile, (b) deformable cell, and (c) box type-foundation with control piles.

Control piles are a special foundation system used to correct differential settlements of buildings and problems related to regional subsidence [1], [2], [3]. The components of the system are a point-bearing pile that freely penetrates the foundation slab, a metallic load frame rigidly anchored to the foundation slab and a deformable cell placed between them (Fig. 1a). The deformable cell is composed of a set of *caobilla* wood cubes arranged in three levels [4], [5] (Fig. 1b). The elastoplastic behavior of the wooden cubes allows transmission of almost constant load to the piles and, in turn, absorption of vertical displacements of the building [6] (Fig. 1c).

### 2.2 Materials

**Fig. 2:**
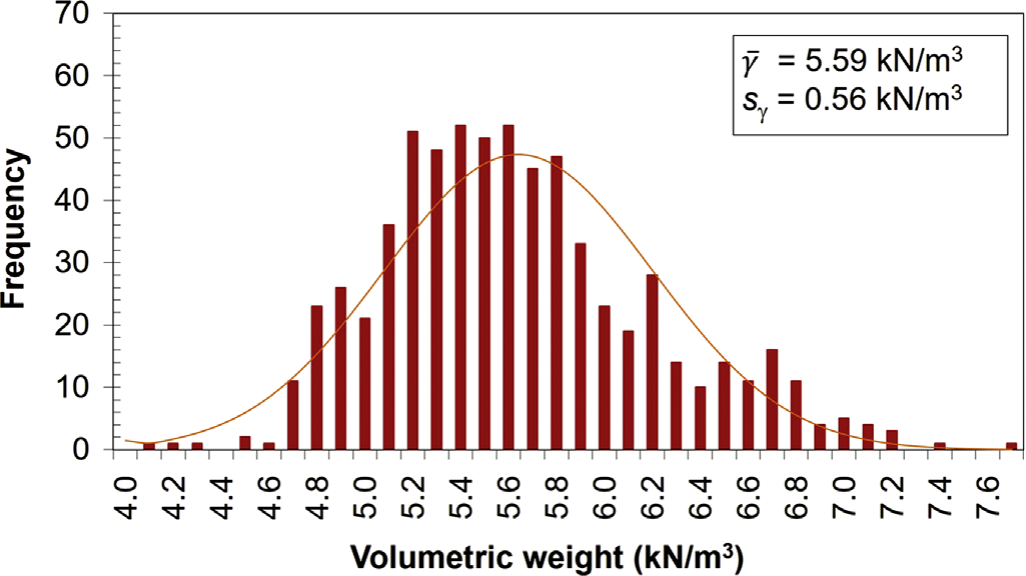
Histogram of the volumetric weight of individual *caobilla* wood cubes.

The *caobilla* wood (*Swietenia humilis Zucc.*) is obtained from trees (15–20 m high) in the tropical dry forest areas of the Mesoamerican Pacific [7]. The wood is dried during 30 days using the Air-Drying method [8]. Afterwards, the wood is cut into cubes of 5 cm edge by means of a Dewalt high precision saw (model DW721KN-B3). Then, the cubes are measured with a Vernier and weighed on a scale. The volumetric weight of the cubes is obtained from the processing of these data. Fig. 2 shows the volumetric weight histogram of 665 wooden cubes (Supplementary Appendix A).

The moisture content MC in the cubes (after air-drying) is analyzed by the Oven-Drying method [9]. This method consists of introducing the specimens (previously weighed) in an oven at a temperature of 103 ± 2 °C. The samples should remain in the oven until an appreciable weight change occurs in 4-h weighing intervals. The moisture content is calculated as follows:

(1) $$\mathrm{MC}\left(\%\right)=\frac{w_{\mathrm{water}}}{w_{\mathrm{wood}}}\left(100\right)$$

where $w_{\mathrm{water}}$ is the weight of water in wood and $w_{\mathrm{wood}}$ is the weight of the oven-dry wood.

### 2.3 Experimental design

**Fig. 3:**
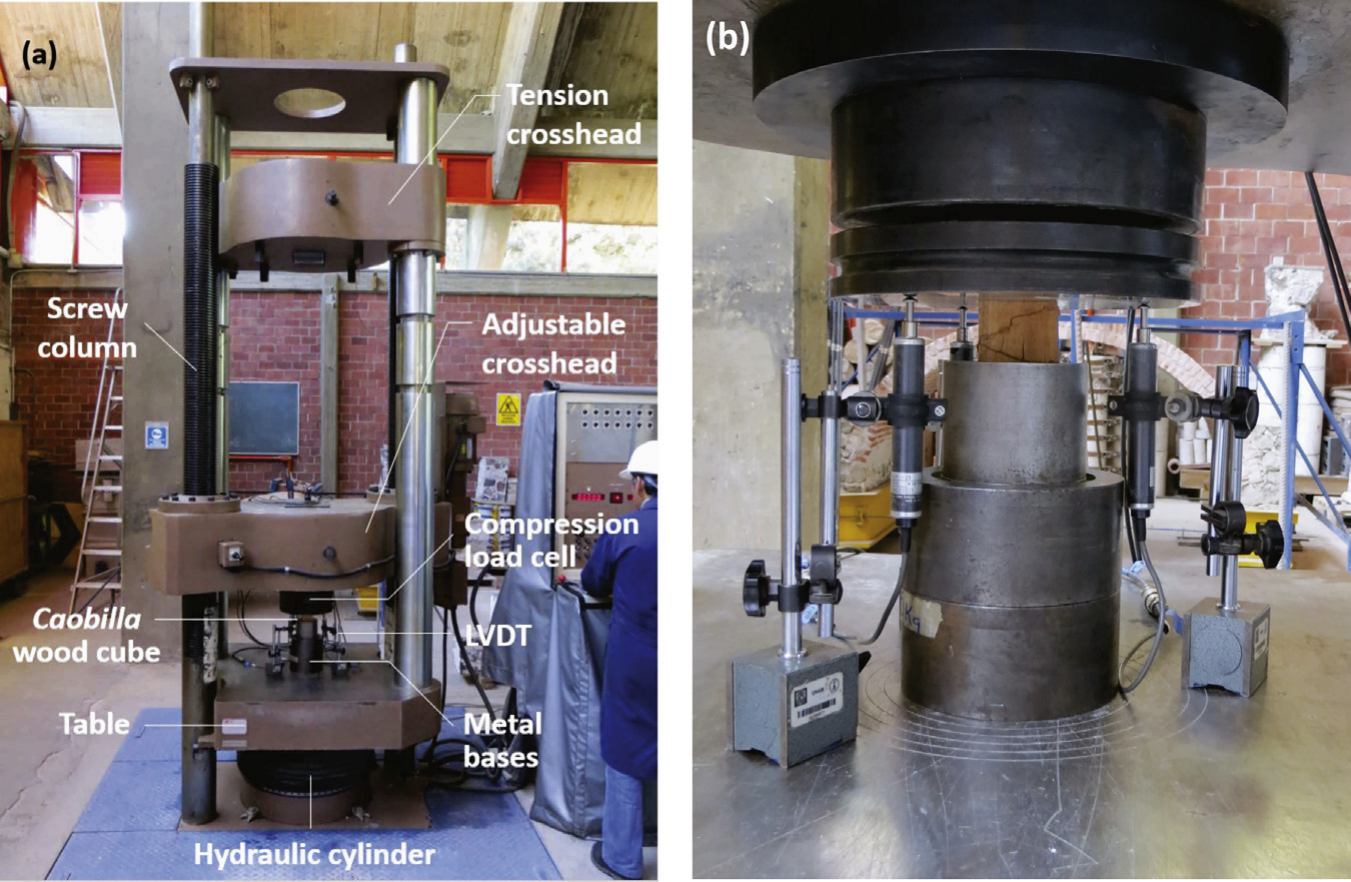
The universal hydraulic machine (vertical compression): (a) details, and (b) instrumented specimen.

The cubes were subjected to stress-controlled compression tests (2 MPa/min) in a Universal Testing Machine (Instron® - SatecTM, model 500 WHVL) with a maximum capacity of 2452 kN (Fig. 3). The wooden cube deformation was measured using three linear variable differential transformers (LVDT, Tokyo Sokki Kenkyujo Co model CDP-25). The data acquisition software allowed the synchronous capture of force and displacement data.

The moisture content of the tested wooden cubes varies between MC = 0.5 to 1% and it represents the “dry state” condition in this article. Additionally, to evaluate the effects of moisture on the mechanical behavior of the wooden cubes, some specimens were submerged in a container full of water for a period of 26 days before being tested. Similarly, in order to assess the influence of the wood anisotropy on the mechanical behavior of the cubes, they were tested with horizontally and vertically oriented fibers. The cubes tested for experimental evaluation were randomly chosen. Table 1 shows the total number of tests performed on individual wooden cubes and their main characteristics.

### 2.4 Methods

**Fig. 4:**
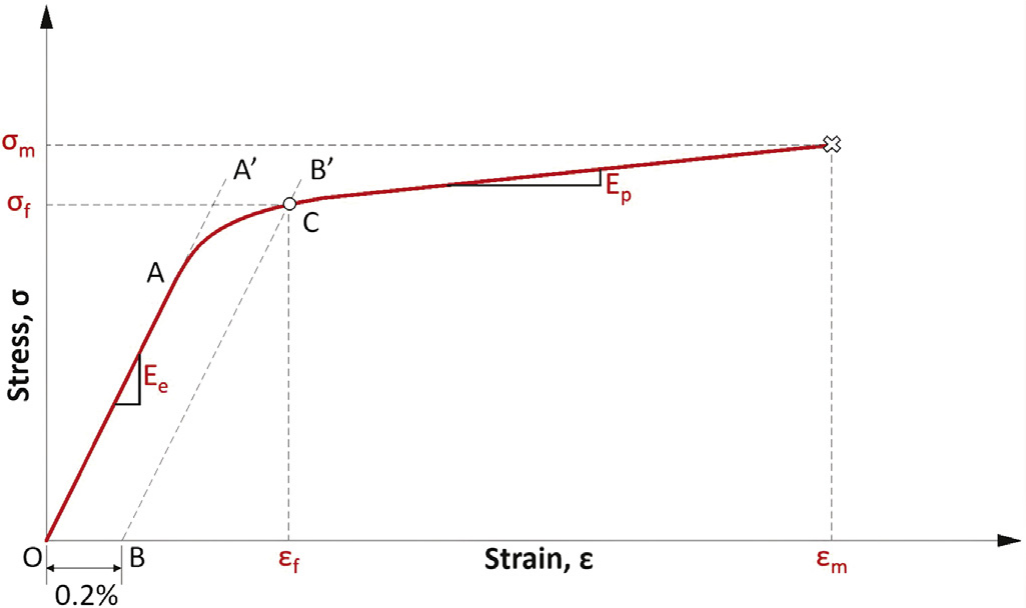
Stress-strain diagram for obtaining the yield stress by the Offset Method [10].

The mechanical properties of the wooden cubes are obtained from stress-strain diagrams, which are generated based on the recorded data in the stress-controlled compression tests. In these diagrams the yield point (yield stress σ_f_) is calculated by the Offset Method [10]. This method consists of drawing a line (O-A′) on the elastic range of the stress-strain diagram. Next, a second 0.2% parallel line to O-A′ must be drawn (line B-B′). The point where line B-B′ intersects the stress-strain diagram (point C) is assumed the yield point (Fig. 4). The yield stress σ_f_ is the stress that produces in a material a specific, permanent and limiting deformation. Similarly, from the stress-strain diagram it is also possible to calculate the tangent modulus in the elastic range E_e_ (modulus of elasticity) and the plastic range E_p_, as well as the maximum stress σ_m_.

### 2.5 Descriptive statistics

**Fig. 5:**
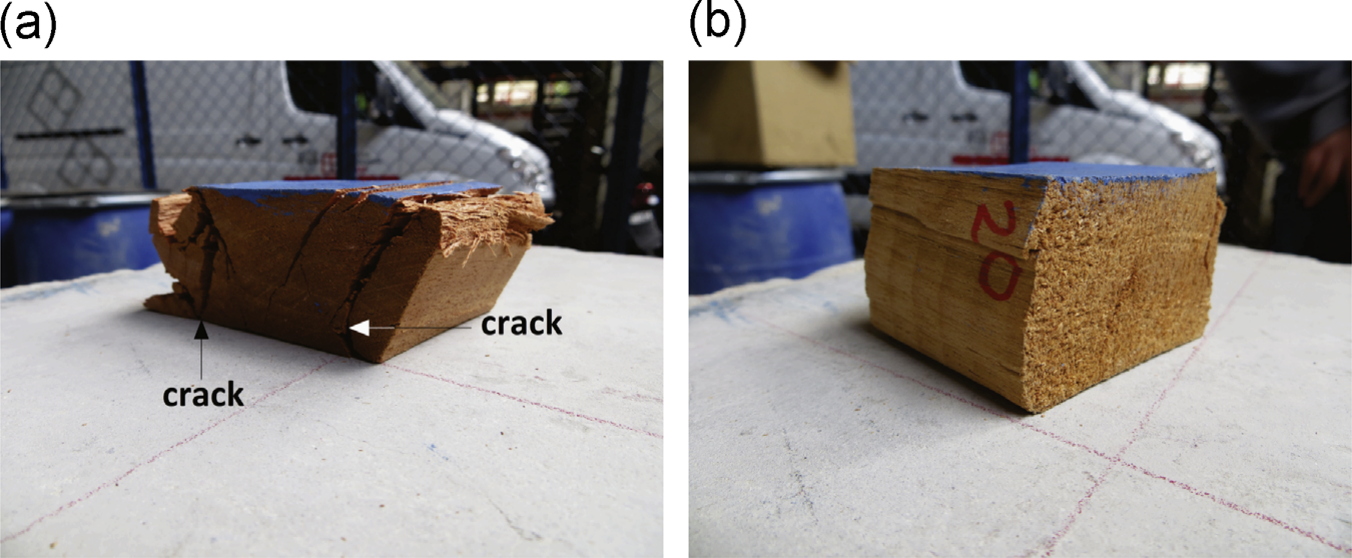
Failure of individual dry cubes of *caobilla* wood with horizontally oriented fibers.

**Fig. 6:**
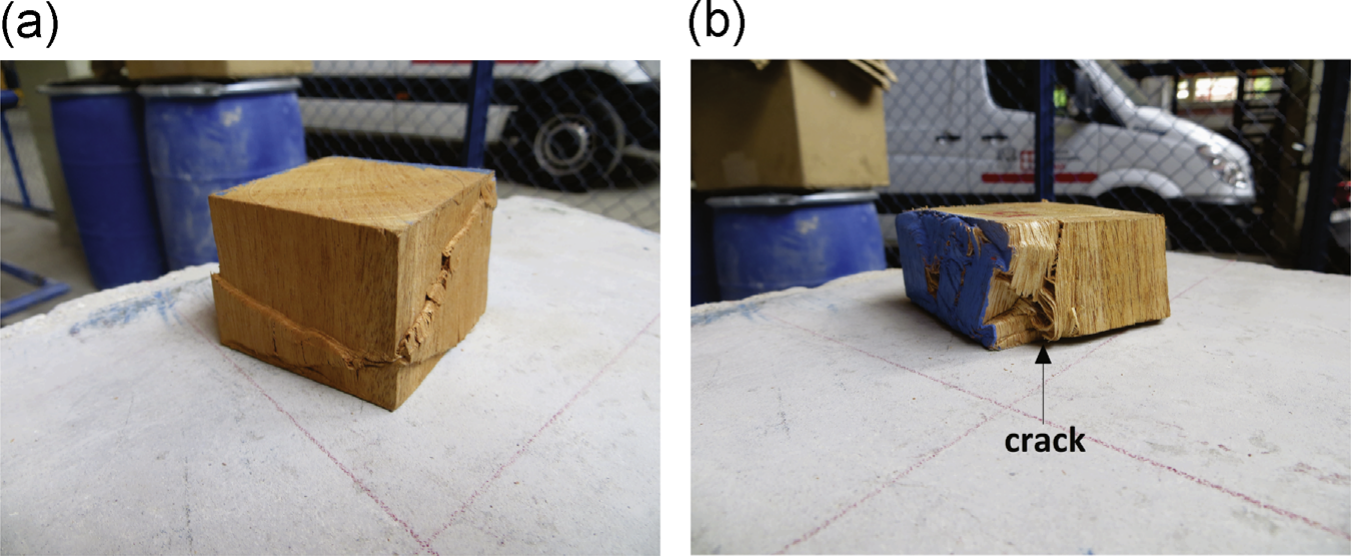
Failure of individual dry cubes of *caobilla* wood with vertically oriented fibers.

**Fig. 7:**
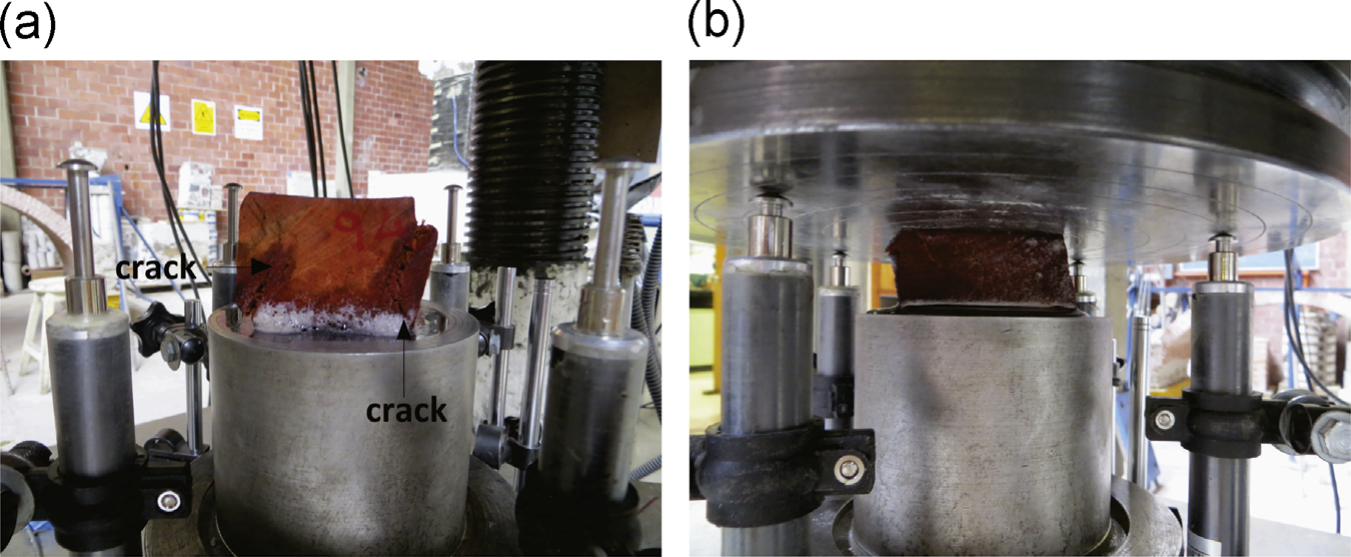
Failure of individual wet cubes of *caobilla* wood with horizontally oriented fibers.

**Fig. 8:**
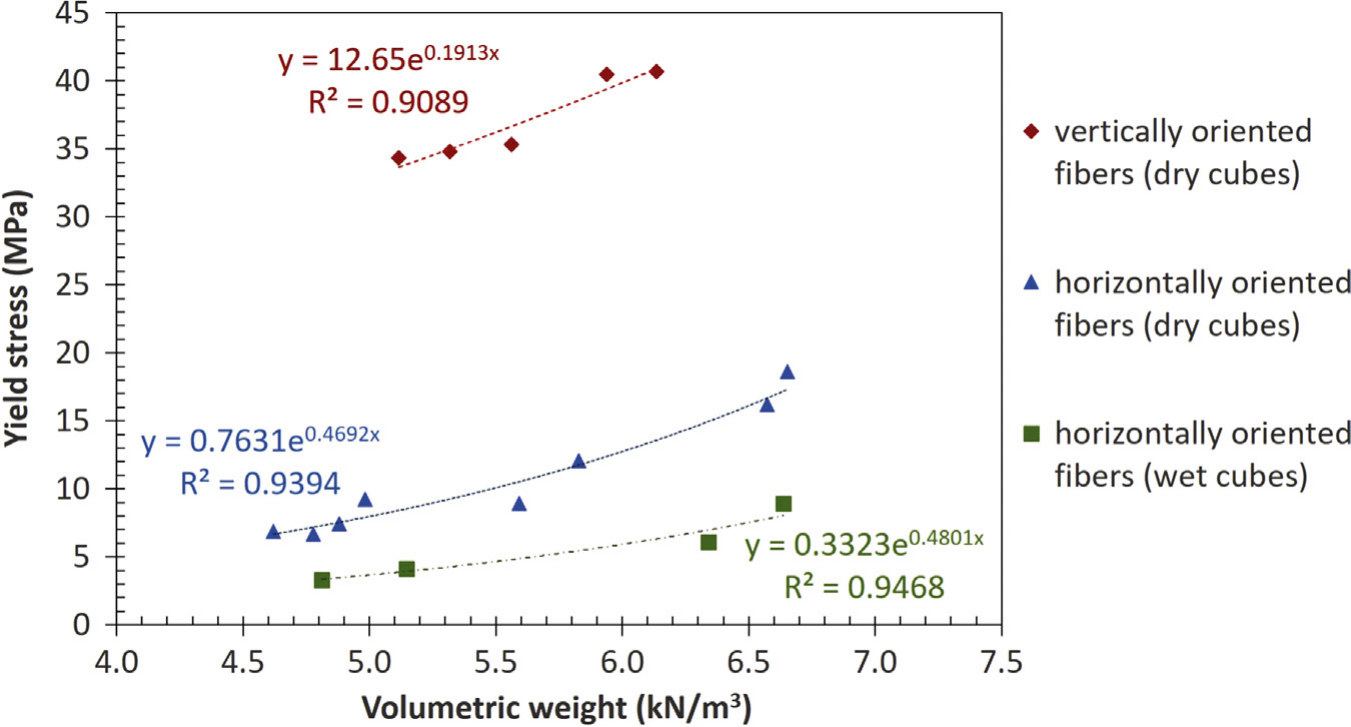
Correlation of yield stress versus volumetric weight of *caobilla* wood cubes.

Table 2 to Table 4 show the data obtained in the compression tests. Table 5 presents the mean values and standard deviations of volumetric weight *γ* and yield stress *σ*_*f*_ of the wooden cubes. Fig. 5, Fig. 6, Fig. 7 show examples of typical failure patterns obtained due to vertical compression on wooden cubes. Fig. 8 presents the correlation of yield stress versus volumetric weight of the wooden cubes (under dry and wet states).

**Table 5:** Mean value and standard deviation of volumetric weight and yield stress of *caobilla* wood cubes.

**Type of specimen**	**Type of test**	$\bar{\gamma }$	**S**_*γ*_	$\bar{\sigma_{f}}$	**Sσ**_**f**_
		**(kN/m**^**3**^**)**	**(kN/m**^**3**^**)**	**(MPa)**	**(MPa)**
Dry cubes	Vertically oriented fibers	5.61	0.42	37.13	3.19
	Horizontally oriented fibers	5.49	0.81	10.75	4.50
Wet cubes	Horizontally oriented fibers	5.73	0.89	5.61	2.50

Note: $\bar{\gamma }$ = mean volumetric weight_*,*_ S_*γ*_ = standard deviation of volumetric weight, $\bar{\sigma_{f}}$ = mean yield stress, Sσ_f_ = standard deviation of yield stress.
